# The Ins and Outs of Autophagic Ribosome Turnover

**DOI:** 10.3390/cells8121603

**Published:** 2019-12-10

**Authors:** Zakayo Kazibwe, Ang-Yu Liu, Gustavo C. MacIntosh, Diane C. Bassham

**Affiliations:** 1 Department of Genetics, Development and Cell Biology, Iowa State University, Ames, IA 50011, USA; zkazibwe@iastate.edu; 2 Roy J Carver Department of Biochemistry, Biophysics and Molecular Biology, Iowa State University, Ames, IA 50011, USA; ayl@iastate.edu (A.-Y.L.); gustavo@iastate.edu (G.C.M.)

**Keywords:** autophagy, lysosome, ribonuclease, ribophagy, ribosome, RNA, target of rapamycin (TOR), vacuole

## Abstract

Ribosomes are essential for protein synthesis in all organisms and their biogenesis and number are tightly controlled to maintain homeostasis in changing environmental conditions. While ribosome assembly and quality control mechanisms have been extensively studied, our understanding of ribosome degradation is limited. In yeast or animal cells, ribosomes are degraded after transfer into the vacuole or lysosome by ribophagy or nonselective autophagy, and ribosomal RNA can also be transferred directly across the lysosomal membrane by RNautophagy. In plants, ribosomal RNA is degraded by the vacuolar T2 ribonuclease RNS2 after transport by autophagy-related mechanisms, although it is unknown if a selective ribophagy pathway exists in plants. In this review, we describe mechanisms of turnover of ribosomal components in animals and yeast, and, then, discuss potential pathways for degradation of ribosomal RNA and protein within the vacuole in plants.

## 1. Introduction

The ribosome is a complex macromolecular machine that is comprised of proteins and RNA. In yeast, it is estimated that ribosomes contain almost 80% of the total cellular RNA and they occupy about 40% of the cytoplasmic volume [1], placing them among the most abundant cellular components. A large body of work has focused on the ribosome assembly and surveillance and quality control mechanisms for both the protein and RNA components that ensure production of functional ribosomes [2]. However, we have a limited understanding of the ribosome turnover process, which involves a tight cooperation between protein degradation and RNA decay and uses mechanisms that span from the cytoplasm to the vacuole or lysosome, and therefore many seemingly contradictory findings need to be reconciled. Here, first, we discuss the turnover of ribosomal components in animals and yeast, and, then, we examine evidence for degradation of ribosomal RNA and protein within the vacuole in plants.

## 2. Degradation of Ribosomes in Yeast and Animals

### 2.1. The RNA Exosome Complex Is Essential for Target of Rapamycin (TOR)- Regulated 25S NRD

**Figure 1:**
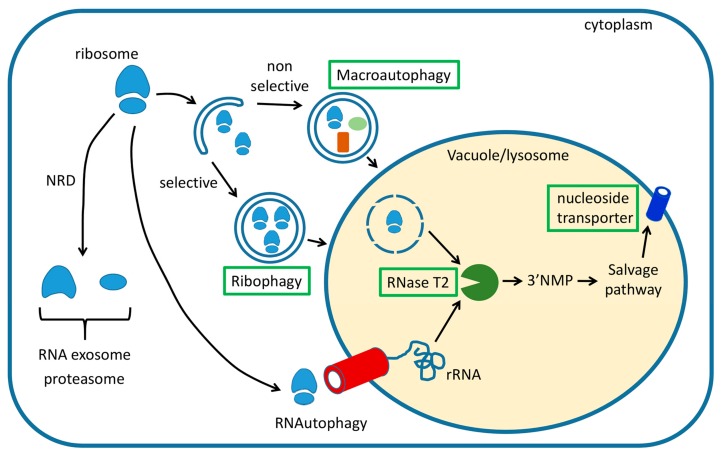
Summary of cellular pathways engaged in degradation of cytoplasmic ribosomes in eukaryotes. Three main pathways have been described, non-functional ribosomal RNA decay (NRD), ribophagy, and nonselective macroautophagy. NRD functions fully in the cytoplasm, while ribophagy and macroautophagy result in the transport of ribosomes to the lytic organelle, where a ribonuclease from the RNase T2 family starts the RNA salvage process. Resulting nucleosides are then transported back to the cytoplasm where they are utilized in cellular metabolism or, in the case of yeast cells, eliminated after further processing. Pathways and components that are believed to function in plants are indicated with green boxes. For details, see main text.

In yeast, non-functional ribosomal RNA decay (NRD) is one of the canonical turnover pathways which degrades ribosomal RNA (rRNA) from ribosomes that are defective in translation (Figure 1) [3,4,5]. NRD is separated into two different pathways which are specific for each ribosomal subunit. The 18S NRD, which is triggered by stalled ribosomes or mutations in the decoding site, degrades 40S ribosomal subunits in a mechanism that requires the cytoplasmic RNA exosome. This process is mediated by the Ski complex, which provides the helicase activity needed to denature and release the 18S rRNA for exoribonuclease cleavage by the exosome-associated 3′ → 5′ exoribonuclease Rrp44 [4,6]. It has also been reported that the cytoplasmic 5′ → 3′ exoribonuclease Xrn1 degrades the 18S NRD substrates [3,7]. The 25S NRD, which is activated by mutations in the peptidyl transferase center [3], degrades the 25S rRNA in a process that has remained more elusive to identify. It has been reported that ubiquitylation of 60S ribosomal proteins by the E3 ubiquitin ligase Mms-Rtt101 complex is the first step in the 25S NRD [8]. The dissociation of 40S subunits is then promoted by the Cdc48 chaperone, a member of the AAA-ATPase family that functions as a segregase, releasing ubiquitylated substrates from the protein complexes for either recycling or proteasomal degradation [9,10,11,12]. The ubiquitylated 60S ribosomal proteins are degraded by the proteasome, leaving the 25S rRNA accessible for further degradation [13]. The RNA exosome complex is presumably the RNase that degrades the unprotected 25S rRNA, but it has also been proposed that the two NRD pathways use different machinery, as 25S rRNA degradation remains unaffected in *ski7*Δ and *xrn1*Δ cells, the two yeast strains carrying 18S NRD null mutations [3].

It has also been reported that the level of functional 25S rRNA may be regulated through a Ski complex-dependent pathway [14] under the control of the protein kinase target of rapamycin (TOR) complex 1 (TORC1), a regulator of many pathways for growth, nutrient, and stress responses [15,16]. The 25S rRNA has been found to be rapidly degraded upon inhibition of TORC1, and the process was severely impaired by knockout of *Ski2* and *Ski4*, two of the members of the Ski complex [14], suggesting the involvement of the RNA exosome in stress-induced ribosome decay unrelated to NRD. This process seems independent of autophagy because the level of 25S rRNA decay remained unchanged in the absence of autophagic proteins (i.e., in *atg* mutants) [14]. However, TOR inhibition results in activation of autophagy [15], and several lines of evidence indicate that normal and stress-induced ribosome turnover is carried out by autophagy-related processes (see below). These contradictory results will need to be resolved by further study.

### 2.2. Ribophagy Is a Selective Pathway that Degrades Mature Ribosomes upon Starvation

Ribophagy, a selective autophagy process specifically targeting ribosome turnover, has been relatively well characterized in yeast and mammals (Figure 1). Triggered by nitrogen deprivation, mature ribosomes are delivered to the vacuole or lysosome in an autophagy-dependent fashion, followed by rapid degradation by vacuolar enzymes [17,18]. Studies in yeast determined that upon amino acid starvation, ribosomal proteins were degraded faster than other cellular proteins and that this process was dependent on the presence of a functional autophagy machinery. Ribophagy is, therefore, a type of selective autophagy that turns over 60S ribosomal proteins. The process also required the ubiquitin protease Ubp3/Bre5 complex [17]. Neither *ubp3*Δ nor *bre5*Δ mutants displayed detectable defects in starvation sensing and general trafficking by macroautophagy, suggesting that the Ubp3/Bre5 complex is involved specifically in selective degradation of large ribosomal subunits through ribophagy [17].

Rpl25 was found to be heavily ubiquitylated in *ubp3*Δ cells [17,19]. Further studies identified Ltn1, a 60S ribosome-associated E3 ligase, as the E3 ligase that modifies Rpl25 and acts as an inhibitor of ribophagy [19]. Knockout of *LTN1* rescued the ribophagy defect in *ubp3*Δ cells, allowing vacuolar transport of GFP-Rpl25 in *UBP3*-null cells [19]. A molecular model of starvation-induced ribophagy has been proposed to illustrate the antagonistic roles of Ltn1 and the Ubp3/Bre5 complex [19]. Under normal growth conditions, the cellular level of Ltn1 is supported by constant translation, counteracting proteasomal degradation. The presence of Ltn1 maintains the ubiquitylation of Rpl25, which is balanced by the deubiquitylation activity of the Ubp3/Bre5 complex. Upon starvation, the rapid decay of Ltn1 moves the balance toward deubiquitylation, triggering the activation of ribophagy and the rapid vacuolar turnover of large ribosomal subunits. The dissection of the ribophagy molecular pathway has been taken one step further with the discovery of a requirement for Cdc48 and its ubiquitin-binding cofactor Ufd3 as crucial components in Ubp3/Bre5-mediated ribosome turnover [20]. Considering its segregase activity in mediating 25S NRD turnover [13], it is reasonable to hypothesize that Cdc48, along with its ubiquitin-binding cofactor Ufd3, partially disassembles mature ribosomes before their enclosure by autophagosomes, either promoting the dissociation of 40S subunits from the mature ribosome or perhaps even the release of ubiquitylated 60S ribosomal proteins from the large ribosomal subunit. Interestingly, the Ubp3/Bre5 complex has been reported to regulate the cytoplasm-to-vacuole targeting pathway through interacting with the receptor Atg19 and, presumably, removing its ubiquitylation, which facilitates vacuolar transport of the Ape1 cargo [21]. Thus, the evidence indicates a common role for the Ubp3/Bre5 complex in selective autophagy through regulation of the ubiquitylation status of the substrates or the receptors.

### 2.3. NUFIP1 Is the Ribophagy Receptor in Mammalian Cells

Selective autophagy receptors recognize specific cargoes and also bind to the autophagic protein Atg8 (LC3 in mammals) to recruit the cargo into forming autophagosomes [22,23]. It is still unknown whether such a receptor is required for ribophagy in yeast, but a candidate ribophagy receptor was recently identified in mammalian cells. Nuclear fragile X mental retardation interacting protein 1 (NUFIP1), along with its partner protein zinc finger HIT domain-containing protein 3 (ZNHIT3), was discovered in quantitative proteomic analysis as one of the proteins whose lysosomal abundance increased upon inhibition of mTORC1 [18]. Further analyses confirmed that NUFIP1, which was previously known as a nucleocytoplasmic shuttling protein regulating the assembly of ribonucleoproteins (RNPs) [24,25,26], redistributes from the nucleus to lysosomes upon mTORC1 inhibition or nutrient deprivation in an autophagosome-dependent manner [18]. NUFIP1 harbors an LC3B-interacting region (LIR), interacts with ribosomes, and is required for delivering ribosomes to autophagosomes for degradation under amino acid deprivation or mTORC1 inhibition [18]. The absence of NUFIP1, or simply the loss of its LIR, impairs cell survival under amino acid starvation [18]. It is not known whether the yeast homolog of NUFIP1, Rsa1, plays a similar role in autophagy-dependent ribosome turnover. However, Rsa1 participates in the regulation of the assembly of small nucleolar ribonucleoproteins and ribosomal subunits, a role conserved among NUFIP1/Rsa1 orthologs throughout eukaryotes [25,27,28,29]. It is possible that its role in ribophagy is also conserved among eukaryotic lineages, and therefore testing its participation in starvation-induced ribophagy in yeast could provide an important advancement in our understanding of this process.

### 2.4. The Selectivity of Ribophagy Has Been Questioned

Meanwhile, the concept of ribophagy as a selective form of autophagy has been challenged by a recent study. An and Harper (2018) established a reporter system in which ribosomal proteins, RPS3 and RPL28, were C-terminally tagged with the fluorescent protein Keima, a dual-excitation reporter that undergoes a switch of chromophore resting charge state upon pH change [30]. This enabled them to monitor ribophagy flux in the human cell lines HCT116 and HEK293 by measuring lysosomal trafficking with the shift of fluorescent emission [30]. Inhibition of translational elongation or nascent chain uncoupling did not activate ribophagic flux, indicating that NRD does not proceed through ribophagy. Alternatively, lysosomal trafficking of Ribo-Keima proteins was successfully promoted by starvation or mTOR inhibition, as previously mentioned [18], and by arsenite or reversine which are compounds that cause proteotoxicity [30]. Treatment with a p97 (Cdc48 in yeast) inhibitor did not inhibit starvation or mTOR inhibition-promoted ribophagy, but the ribophagy induced by arsenite did require a functional p97 [30]. Their study also found differences in ATG5 requirement between ribophagy of RPL28-Keima and RPS3-Keima [30], resembling previous results indicating that 60S and 40S ribosomal subunits are targeted for autophagic degradation separately and with distinct regulation in yeast [17]. Importantly, this work showed that in human cells, amino acid starvation and mTOR inhibition promote ribophagic flux at the same rate as that observed for other cytosolic proteins, and thus the process is not selective. Systematic analysis indicated that other forms of selective autophagy result in “bystander flux” that leads to increased rates of ribosome and cytosolic protein lysosomal degradation [30]. The results of this study should perhaps be re-evaluated in light of a report indicating that starvation induces rapid turnover of selective autophagy receptors in human cell lines by a microautophagic process [31]. In any case, the study by An and Harper confirms that ribosomes are included in the cytoplasmic materials destined for autophagic degradation upon stress stimulation.

A comprehensive study of bulk RNA (primarily consisting of rRNA) degradation via nitrogen starvation-induced autophagy in yeast also showed inconsistencies in the proposed mechanisms for vacuole-dependent RNA degradation and the selectivity of ribophagy. A detailed characterization of the RNA salvage pathway in yeast suggested that starvation promotes autophagy-dependent bulk RNA turnover, which largely depends on the core macroautophagy machinery but is independent of the ribophagy-specific machinery such as the Ubp3/Bre5 complex (Figure 1) [32]. The vacuolar acid ribonuclease Rny1, the sole member of the RNase T2 family in yeast [33], has been shown to catalyze the first step in RNA recycling after autophagic transport to the vacuole, generating 3′ nucleoside monophosphates (3′ NMP), which are then processed by the vacuolar phosphatase Pho8 to produce nucleosides as the final vacuolar product [32]. Microscopic observation indicated that RNA is transported to the vacuole in both *ubp3*Δ and *bre5*Δ cells upon starvation, and similar levels of intracellular 3′ NMP were observed in Ubp3/Bre5-deficient cells as in wild-type yeast, suggesting that the deubiquitylation complex does not participate in delivering RNA to the vacuoles for degradation upon starvation.

A slightly delayed time course of 3′ NMP generation was indeed observed in Ubp3/Bre5-deficient cells, with a slower conversion to the downstream nucleosides. However, due to an observed partial defect in the levels and activities of vacuolar enzymes, the authors suggested that the Ubp3/Bre5 complex, rather than participating in cargo sequestration, is involved in membrane trafficking, supporting the high membrane demands during starvation-induced autophagy [32]. Indeed, the Ubp3/Bre5 complex has long been known to regulate coatomer protein complex (COP) II and COP I-dependent vesicle transport between the ER and Golgi [34,35], a process known to be closely connected to autophagosome biogenesis [36]. Deciphering the connection between starvation-induced bulk RNA degradation and Ubp3-dependent ribophagy should be an interesting goal to extend our understanding of vacuole-dependent ribosome turnover.

### 2.5. Direct Uptake of Nucleic Acids by Lysosomes: RNautophagy and DNautophagy

A novel type of autophagy in which RNAs, as well as DNAs, are directly imported into lysosomes for degradation has recently been described in animal cells, and this process has been named RNautophagy and DNautophagy (Figure 1) [37,38]. The pathway requires two lysosomal membrane proteins, LAMP2C and SIDT2, to directly take up nucleic acids for lysosomal degradation [37,38]. In concept, this resembles the direct transport of proteins across the lysosomal membrane by chaperone-mediated autophagy (CMA), which requires LAMP2A as a receptor and HSPA8/HSC70 as a chaperone to unfold and translocate the targeted proteins into the lysosome individually [39,40,41]. RNautophagy is capable of transporting rRNA into lysosomes and requires LAMP2C, a different splice variant of the lysosomal transmembrane protein LAMP2 than that required for CMA. LAMP2C interacts directly with both RNAs and DNAs through its specific cytosolic tail [37,38] and the 11 amino acids of the cytosolic domain of LAMP2C were also shown to indirectly associate with a variety of RNA-binding proteins in RNA-mediated interactions [38]. In vitro experiments with isolated lysosomes showed that RNA uptake and degradation are increased by overexpression of LAMP2C and impaired by LAMP2 knockout, and that the transport process needs ATP [37,38]. The level of total RNA in brain tissues was found to be higher in LAMP2 knockout mice as compared with the WT control [38]. In later studies, SIDT2, the ortholog of the *Caenorhabditis elegans* putative RNA transporter SID-1, was also reported to be a lysosomal membrane receptor contributing to lysosomal uptake and degradation of nucleic acids [42,43]. Although protein–protein interaction was demonstrated between SIDT2 and LAMP2C, the two receptors are believed to work independently, because SIDT2 overexpression increased RNA uptake in the absence of LAMP2 [43].

### 2.6. Physiological Roles of Vacuole- and Lysosome-Dependent RNA Turnover

In addition to identifying autophagy as an important pathway for RNA turnover, recent advances have shown that this process helps to maintain cellular homeostasis through RNase T2 family-dependent RNA hydrolysis in different model systems. The metabolic networks affected by this RNA turnover have been revealed by recent studies, and the pathophysiological consequences of defects in the process have been demonstrated by molecular and genetic studies, both in model organisms as well as in human case studies.

In yeast, after vacuolar import of RNA by autophagy, triggered by starvation, the vacuolar T2 ribonuclease Rny1 launches the degradation process by RNA hydrolysis (Figure 1), generating 3′ NMP products that are then converted to nucleosides by the vacuolar phosphatase Pho8 [32]. Through nucleoside transporters such as Fun26 [44], the intermediates are translocated back to the cytoplasm where further conversions are conducted by two nucleosidases, Pnp1 and Urh1 [32,45,46]. The nucleobases produced in the cytoplasm undergo further catabolic conversions into the final products, xanthine and uracil [32]. Interestingly, the majority of product released from vacuole-degraded RNA is secreted to the medium in the form of xanthine and uracil with only a transient accumulation in the cytoplasm. This indicates that vacuole-dependent RNA degradation is unlikely to be a salvage pathway replenishing nucleotide synthesis during starvation in yeast [32]. Indeed, *Saccharomyces cerevisiae* is unable to utilize purines and pyrimidines as nitrogen sources due to the lack of specific metabolic enzymes [47]. A proper disposal system which excretes RNA degradation products could be important to maintain cellular homeostasis during nitrogen starvation in this model system.

In contrast, lysosome-dependent RNA degradation serving as a salvage pathway has been found to be critical to *Caenorhabditis elegans* development. The T2 ribonuclease RNST-2 was recently identified as the lysosomal enzyme in *C. elegans* responsible for rRNA hydrolysis. Its activity serves as a key regulator of nucleotide homeostasis in *C. elegans* [48]. At the cellular level, *rnst-2* mutants bear enlarged lysosomes that accumulate undigested rRNA and ribosomal proteins, and this accumulation is autophagy-dependent. Mutants have lower viability with defects in embryogenesis and larval development, and these defects can be partially ameliorated by uridine or cytidine supplementation [48].

In the zebrafish *Danio rerio*, two genes encoding T2 ribonucleases have been described to be orthologues of human *RNASET2*, *rnaset2l* (ribonuclease T2 like) and *rnaset2* [49,50]. Haud et al. showed that mutations in *rnaset2* lead to 28S rRNA accumulation in lysosomes of brain neurons, which are enlarged in mutant fish, and this cellular phenotype resulted in a new type of lysosomal storage disorder caused by deficient RNA turnover [49]. White matter lesions with dilated ventricles were found in the mutants [49], resembling the symptoms of familial cystic leukoencephalopathy arising in *RNASET2*-deficient humans [49,51]. These results demonstrated the essential housekeeping role of T2 ribonucleases in lysosome- and vacuole-dependent RNA salvage and nucleoside homeostasis.

## 3. Degradation of Ribosomes in Plants

As in other organisms, the number and quality of plant ribosomes must be regulated for proper translation and homeostasis [52,53]. While ribosome synthesis and assembly is relatively well studied [53,54,55,56,57], little is known about the turnover of mature ribosomes at the end of their useful life. As in other organisms, mature plant ribosomes can be degraded to facilitate recycling of building blocks such as nucleotides and amino acids for use in primary metabolism to either maintain cell homeostasis or in response to stress. While the RNA exosome has been well characterized in plants [58,59,60,61], there is no evidence supporting the existence of an NRD pathway. Thus, in this section, we discuss the mechanisms by which ribosomes, or their constituents, are transported to and degraded inside plant vacuoles and the involvement of autophagy in this process. We also discuss the consequences of defects in the ribosome and RNA decay pathway on the physiology of plants and possible future directions in plant ribophagy research.

### 3.1. Evidence for RNA Degradation in Plant Vacuoles

In plants, canonical ribophagy has not been demonstrated but rRNA turnover in *Arabidopsis thaliana* is known to occur at least partially within vacuoles. The ribonuclease RNS2 is necessary for normal ribosomal RNA decay in Arabidopsis (Figure 1) [62]. RNS2 is a nonspecific endoribonuclease of the RNase T2 family that localizes to the vacuole and endoplasmic reticulum [62,63]. During senescence and phosphate starvation, RNS2 is upregulated, possibly to remobilize the phosphate in RNA [64,65]. By contrast, reduced RNase activity is seen in maize roots under low oxygen conditions, correlating with low RNA or ribosome turnover [66], suggesting that under low oxygen conditions, RNase activity, likely including RNS2, is reduced to conserve RNA. Together, these findings suggest that ribonuclease activity is highly regulated in different environmental conditions in order to maintain cellular homeostasis [64,65,66].

Arabidopsis mutant plants lacking RNS2 (*rns2-2*) accumulate RNA inside the vacuole [67], and rRNA has a longer half-life in these mutants as compared with wild-type plants [62]. This suggests that rRNA must be delivered from the cytoplasm to the vacuole for degradation by RNS2, potentially by a ribophagy-like process. Additionally, the *rns2-2* mutant plants have constitutive autophagy [62,67], possibly as an attempt to compensate for loss of rRNA degradation. The localization of RNS2 to the vacuole is critical for normal ribosomal RNA turnover and homeostasis [63], as shown using a mutant (*rns2-1*) that lacks the C-terminal vacuolar targeting signal and is instead secreted from the cell. Although it retains RNase catalytic activity, *rns2-1* has constitutive autophagy, similar to the null mutant *rns2-2* [62,63]. This indicates that disruption of vacuolar localization leads to a loss of the physiological function of RNS2, which can no longer participate in ribosomal RNA turnover in the vacuole [63].

After degradation, the rRNA breakdown products in the vacuole need to be exported back to the cytoplasm for reuse by the cell. Similar to the equivalent enzyme Rny1 in yeast [32], Arabidopsis RNS2 may degrade RNA to 3′-nucleotide monophosphates (3′NMPs), which may then be dephosphorylated by an as yet unidentified nucleoside phosphatase in the vacuole. The resulting nucleosides can then be transported by the Arabidopsis tonoplast equilibrative nucleoside transporter 1 (ENT1) [68] to the cytoplasm, where they may be converted to purine bases and subsequently used in primary metabolism. Vacuolar degradation of rRNA as part of the RNA salvage pathway is expected to be important in nucleotide homeostasis and cellular metabolism (Figure 1).

### 3.2. Physiological Effects of Defects in Vacuolar rRNA Degradation

The constitutive autophagy seen in *rns2* mutants has been hypothesized to be due to changes in cellular homeostasis and lack of normal RNA decay, leading to reduced cytoplasmic nucleoside concentrations [62,63,67]. A comparison of gene expression profiles in *rns2-2* mutants as compared with wild-type plants [69] indicated that, surprisingly, very few genes were differentially expressed. Among the upregulated genes in *rns2-2*, genes encoding pentose phosphate pathway (PPP) enzymes were overrepresented. In addition, the levels of some PPP intermediate metabolites were significantly reduced in *rns2-2*, suggesting changes in flux through the pathway [69]. These changes suggested that the oxidative phase of the PPP is repressed in *rns2-2*, while the non-oxidative phase is activated to favor the production of ribose-5-phosphate from glyceraldehyde-3-phosphate and fructose-6-phosphate. Carbon flux can, therefore, be diverted via the PPP toward the production of ribose-5-phosphate, which is an essential substrate in de novo purine synthesis, to compensate for the loss of recycling of nucleosides [69,70].

The mechanism by which basal autophagy is enhanced in *rns2-2* is yet unknown. Autophagy is upregulated during nutrient starvation, and is involved in recycling of macromolecules to alleviate nutrient deficiency [71,72]. The mammalian target of rapamycin protein kinase complex (mTORC1) has been reported to be involved in sensing purine nucleotide levels, to coordinate downstream responses such as nucleic acid synthesis [73]. We speculate that Arabidopsis target of rapamycin (TOR) may detect reduced nucleotide levels in the cytoplasm of *rns2-2* plants, resulting in reduced TOR activity and, consequently, activation of autophagy, since TOR negatively regulates autophagy [74,75]. Measuring TOR activity in the *rns2-2* mutant could help to test this hypothesis.

The *rns2-2* mutant has elevated levels of reactive oxygen species (ROS) [69], possibly resulting from increased production of NADPH by the PPP [69] leading to increased NADPH oxidase activity. The accumulation of ROS in plants induces autophagy [76,77], and therefore elevated levels of ROS may alternatively explain the constitutive autophagy of *rns2-2* mutant. This constitutive autophagy is blocked by the addition of DPI, an inhibitor of NADPH oxidase, suggesting that in *rns2-2*, autophagy is activated by an NADPH oxidase-dependent pathway, supporting this hypothesis [69].

Phenotypic analysis of the *rns2-2* mutants revealed that they are larger than the wild type. This may be due to observed increases in expression of both cell wall enzymes such as glycosyl hydrolases, as well expansins, and a decrease in the monosaccharides mannose and glucuronic acid that could cause reduced crosslinking of hemicellulose [78]. Elevated levels of cell wall enzymes facilitate cell wall elongation [79,80], while high levels of expansins result in loosening of cell walls [80], allowing greater cell expansion and increased cell size through water influx. These morphological changes could be a secondary consequence of rerouting carbon sources. Taken together, these findings suggest a model in which loss of RNS2 activity and RNA degradation results in reduced nucleotide levels, triggering changes in carbon flux and activating autophagy to provide carbon backbones to be used in the PPP for de novo nucleoside synthesis.

### 3.3. Are Entire Ribosomes Degraded in the Vacuole, or Just RNA?

Ribosomes are composed of proteins and RNAs at a weight ratio of approximately 3:2 in plants [81,82], indicating that the degradation of ribosomes is a significant source of amino acids from ribosomal proteins and nucleosides and nucleobases from rRNA. In plants, it remains to be determined whether intact ribosomes are transferred to the vacuole for degradation, or if ribosomes are first disassembled in the cytoplasm. As described, in yeast, mature ribosomes are targeted to the vacuole for degradation by ribophagy and nonselective autophagy [17,32]. The extent to which ribophagy occurs in plants is not clear and the stimuli inducing selective ribosome turnover are not known. It is possible that, in plants, nutrient deprivation, especially nitrogen starvation, may lead to ribosome degradation to recycle amino acids or nucleobases for primary metabolism. Recycling of these nutrients may be critical for normal cellular metabolism and homeostasis, not only during starvation but also during normal growth conditions [62,67], even though ribosome and RNA degradation may be upregulated during environmental stress [83,84].

Some evidence exists for the transfer of intact ribosomes to the vacuole in plants. In maize root apical meristems, dense aggregates of polyribosomes and rough endoplasmic reticulum first, form in the cytosol, then, invaginate into an adjacent vacuole, and subsequently they are degraded [85], even under normal growth conditions. These granules are mainly composed of RNA and protein suggesting, at least in these conditions and cell types, that whole ribosomes can be transferred for degradation in the vacuole. Ribosomes attached to the ER are also degraded in the vacuole during ER stress [76].

It is possible that a mechanism of direct RNA transport to the vacuole, analogous to RNautophagy, could also exist in plants, for which disassembly of rRNA from ribosomal proteins prior to transport would be necessary, but evidence for this awaits further research. As LAMP proteins are absent from plant genomes (our observation), direct RNA transport to vacuoles in plants would require a different mechanism than that in animals.

### 3.4. The Role of Autophagy in Ribosome Turnover

In Arabidopsis, autophagy is upregulated during environmental stresses, such as nutrient starvation, salt, and drought conditions [86,87]. In addition to targeting individual proteins, both membrane-bound organelles including mitochondria [88,89], chloroplasts [90,91], peroxisomes [92,93,94] and endoplasmic reticulum [76], and non-membranous cellular structures such as the proteasome [95,96], are targeted for degradation in response to environmental cues. Similarly, ribosomes could also be targeted for degradation by autophagy-like mechanisms. Analysis of Arabidopsis *atg* mutants [67] revealed that an autophagy-related mechanism participates in rRNA turnover by RNS2. This pathway is dependent on *ATG5* but not *ATG9* [67], suggesting the existence of a non-canonical autophagy-like process, for example microautophagy, by which ribosomes are degraded. A selective autophagy receptor for ribosomes, NUFIP1, has been reported in mammals [18]. A plant homolog exists and the Arabidopsis protein, AtNUFIP, is essential for the normal assembly of small nucleolar ribonucleoproteins [29], a function also carried out by mammalian NUFIP1 (see above). However, the sequence conservation between plant and mammalian NUFIP proteins is limited to a short conserved motif (PEP) that mediates the interaction with a core C/D snoRNP protein [28,29], and AtNUFIP lacks the LIR region that, in mammals, mediates interaction with LC3B (our observation). Thus, it is not known if AtNUFIP can act as a ribophagy receptor. Other plant organelles can be selectively targeted for degradation, most likely dependent on selective autophagy receptors. Whether such proteins and receptors are involved in plant ribophagy needs further research; the presence of intact ribosomes inside plant vacuoles [76] suggests that they could be targeted by ribophagy receptors. Additionally, Arabidopsis *atg* mutants show increased accumulation of specific proteins including the ribosomal subunits S6 and L13 [29,97], suggesting a role for autophagy in ribosomal protein, as well as rRNA, degradation. How ribophagy-like processes in plants affect the ribosome pool, protein synthesis, and homeostasis is not yet well studied. This may be revealed by genome-wide analyses such as proteomic and genetic approaches to study the nature and composition of the ribosome pool in *rns2-2* or mutants involved in ribophagy-like processes.

## 4. Conclusions

In plants, as in other organisms, the regulation of ribosome quality and number is essential for cell homeostasis, to maintain proper translation, and to respond to environmental stresses. Ribosomes can be degraded by nonselective macroautophagy, but evidence from animal and yeast studies points to the existence of degradation pathways that specifically target ribosomes, termed ribophagy. Despite their potential role and importance in plant physiology and metabolism, ribophagy-like processes, their regulation, and mechanisms in plants remain largely unstudied. Additionally, the fate of ribosomal proteins, rates of ribosome turnover, and ribophagy receptors in plants are unknown. Further research could reveal novel mechanisms of ribosome turnover in plants that are dependent on or independent of *ATG* genes and elucidate the role of such pathways in plant cell homeostasis.
